# Turning Back the Clock: A Retrospective Single-Blind Study on Brain Age Change in Response to Nutraceuticals Supplementation vs. Lifestyle Modifications

**DOI:** 10.3390/brainsci13030520

**Published:** 2023-03-21

**Authors:** Andrew A. Fingelkurts, Alexander A. Fingelkurts

**Affiliations:** BM-Science—Brain and Mind Technologies Research Centre, 02601 Espoo, Finland

**Keywords:** chronological age (CA), biological age (BA), quantitative electroencephalography (qEEG), brain biological age (BBA), nutraceuticals, lifestyle, intervention

## Abstract

Background: There is a growing consensus that chronological age (CA) is not an accurate indicator of the aging process and that biological age (BA) instead is a better measure of an individual’s risk of age-related outcomes and a more accurate predictor of mortality than actual CA. In this context, BA measures the “true” age, which is an integrated result of an individual’s level of damage accumulation across all levels of biological organization, along with preserved resources. The BA is plastic and depends upon epigenetics. Brain state is an important factor contributing to health- and lifespan. Methods and Objective: Quantitative electroencephalography (qEEG)-derived brain BA (BBA) is a suitable and promising measure of brain aging. In the present study, we aimed to show that BBA can be decelerated or even reversed in humans (N = 89) by using customized programs of nutraceutical compounds or lifestyle changes (mean duration = 13 months). Results: We observed that BBA was younger than CA in both groups at the end of the intervention. Furthermore, the BBA of the participants in the nutraceuticals group was 2.83 years younger at the endpoint of the intervention compared with their BBA score at the beginning of the intervention, while the BBA of the participants in the lifestyle group was only 0.02 years younger at the end of the intervention. These results were accompanied by improvements in mental–physical health comorbidities in both groups. The pre-intervention BBA score and the sex of the participants were considered confounding factors and analyzed separately. Conclusions: Overall, the obtained results support the feasibility of the goal of this study and also provide the first robust evidence that halting and reversal of brain aging are possible in humans within a reasonable (practical) timeframe of approximately one year.

Death is […] not an absolute necessity essentially inherent in life itself.

([1], p. 26)

## 1. Introduction

For millennia humans have been fascinated by the prospect of living forever. This aspiration has left noticeable marks in virtually every human culture reflecting on the possibility of transcending death [2,3]. While such an extreme wish to attain some form of immortality is still implicitly embedded in the so-called movement of “posthumanism” (*posthumanism seeks to improve human nature by using technology to transcend the limitations of the body and mind [4,5,6]*) (for a brief overview, see [7]), in biomedical science it has been transformed into a more practical aim of slowing down or potentially even reversing aging [8,9,10,11,12], progressively reaching the “age escape velocity” (*such an approach presupposes that death could be interactively delayed by anticipating and fixing the damaging effects of aging across the lifespan [13]*), which will open the prospect of extreme human life extension [14].

**Figure 1:**
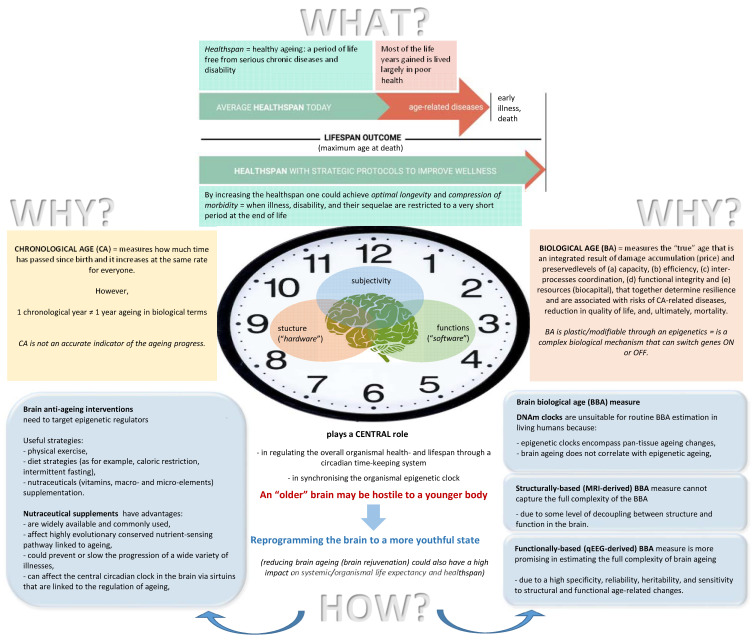
Introduction: What? Why? and How?

Over the past half century, life expectancy and the observed maximum age at death have increased dramatically [15], probably due to the successes of evidence-based medicine, which have been very effective at reducing mortality over the past few decades [16]. At the same time, it has become painfully evident that not all of the gained extra years are healthy: estimates have shown that the proportion of life characterized by good health has remained rather constant between 1990 and 2019 [17], implying that most of the life years gained are lived largely in poor health [12]. As pointed out by Olshansky [18], this leads to a situation where a significant portion of the lifespan is lived during a window of exponentially increasing risk of frailty and chronic disability (Figure 1), with the simultaneous manifestation of many chronic conditions as late life comorbidities [16,19,20]. Therefore, there is an increasing understanding of the importance of so-called “healthy aging” (*healthy aging refers to the “healthspan”, which is a period of life free from serious chronic diseases and disability [21]. It has been proposed that by increasing the healthspan, one could achieve optimal longevity, when illness, disability, and their sequelae would be restricted to a very short period at the end of life—termed “compression of morbidity” [22]. Such optimal longevity would signify entering a fourth stage of epidemiological transition according to Omran [23]—the age of delayed degenerative diseases [24]*) [21,25] and an unprecedented advance in research that focuses on the biology of aging [9,11,26,27].

Aging is commonly characterized as a progressive loss of physiological function due to the accumulation of molecular and cellular damage, leading to the development of chronic comorbidities that include metabolic, immune, cardiovascular, neoplastic, and neurodegenerative disorders, accompanied by geriatric symptoms, such as frailty and immobility [28,29,30]. Over the past few decades, some of the mechanistic pathways involved in aging have been elucidated; they are known as mechanisms [31], principles [32], biomarkers [33], hallmarks [34], pillars [35], or predictors [36] of aging. While the actual number of these hallmarks varies depending on the authors, the total is nine [34]: (1) genomic instability, (2) epigenetic alterations, (3) loss of proteostasis, (4) deregulated nutrient sensing, (5) mitochondrial dysfunction, (6) cellular senescence, (7) stem cell exhaustion, (8) altered intercellular communication, and (9) telomere attrition (recently, three additional hallmarks were added: chronic inflammation, disabled macroautophagy, and dysbiosis). Although the contribution of each of these hallmarks to the progression of aging is far from being completely understood (*for a critical discussion of the hallmarks of aging, see Gems and de Magalhães [37]*), it is nevertheless clear that they are interconnected and play a significant causal role in the process of aging [30].

When speaking about age, two concepts are sometimes used interchangeably, but they nonetheless have to be distinguished [29]: *chronological age (CA)* and *biological age (BA)* (Figure 1). Until recently, CA was a commonly used indicator of aging [38] as a universal feature shared by all living beings [16]; however, it only measures how much time has passed since birth, and it increases at the same rate for everyone [39,40]. CA has been shown to be a strong predictor of health status and mortality [19]. At the same time, life expectancy shows considerable variation among individuals with an equal or similar CA [38]. This means that if, for example, one chronological year has passed, it does not necessarily mean that an individual has also aged in biological terms the equivalent of one year [39,41]. It seems that the speed of aging processes varies both between different people [36,42], even in twins [41], and also within the same individual at different periods of the lifespan—the fluidity of ageotypes [42] (see also [43,44]). Therefore, CA is not an accurate indicator of the aging progress [45].

These inter- and intraindividual differences in aging can be captured by BA [35,46,47], which is thought to measure an individual’s risk of age-related outcomes and predict mortality better than actual CA [36,48]. In this context, BA (being a quantitative phenotype [29]) measures the “true” age (*multiple longitudinal studies have shown that BA is the most convenient and reliable measure to determine the extent of age-related (i.e., biomarker) changes in an organism [29]. In this context, higher BA values are indicative of a higher intensity of age-related detrimental processes in comparison with CA, while lower BA values are proxy markers of a lower intensity of aging processes and overall higher resilience to them. Traditionally, BA metrics are designed to resemble the CA distribution within a cohort of healthy individuals, however, being more predictive of a person’s health status than CA itself [49]*), which is an integrated result of an individual’s level of damage accumulation (i.e., *price*) at all levels of biological organization and preserved (a) capacity (i.e., maximal processing power), (b) efficiency (i.e., minimum number of operations and the energy expenditure per operation), (c) interprocess coordination, (d) functional integrity, and (e) resources (i.e., *biocapital*) which together determine resilience (i.e., compensatory and recovering mechanisms) and are associated with the risks of CA-related diseases, vigilance and cognitive decline, reduction in quality of life, and, ultimately, mortality [19,21,50,51,52,53,54,55] (Figure 1).

Continuously growing data suggest that variability in the BA process is due to the diversity in genotypes (i.e., longevity or senescent mutations), family history (*for example, having long-lived parents and grandparents is strongly correlated with a longer lifespan [56]*), lifestyle habits (e.g., smoking, alcohol/drugs consumption, type of diet, physical and mental/intellectual exercise, duration and quality of sleep, medication use, occupational complexity, leisure activity, and social engagement), and environments that include (i) early-life development (i.e., utero characteristics and early stress/trauma), (ii) socioeconomic status, (iii) education level, and (iv) malnutrition, vitamins, and/or nutrient deficiencies or imbalances [39,56,57,58,59,60,61,62,63,64,65,66,67,68,69,70,71,72]. Therefore, BA is plastic and hinges on the balance between the factors mentioned above [73,74].

This interaction of genotype with living habits and the environment is known as *epigenetics* [75]. Epigenetics is a complex biological mechanism that can switch genes ON or OFF, e.g., sleep, diet, and exercise can all cause chemical modifications around specific genes (*DNA methylation is one of the major classes of epigenetic modifications in which a methyl group (one carbon) is covalently added to the C5 position of a cytosine base [76]. The degree of DNA methylation defines gene expression. The other classes include histone modifications and chromatin remodeling [77]*) and histone proteins, hence, either promoting or silencing their expression over time and even leading to heritable changes to the genome without changes to the DNA sequence itself [77]. Accordingly, epigenome changes have consequences for the molecular pathways of cells, tissues, and organs [78]. Increasing empirical evidence demonstrates that certain changes in the epigenome during aging lead to genomic alterations and instability, contributing to the initiation of age-related diseases, such as cancer and neurodegenerative diseases (*interest in epigenetic mechanisms is increasing due to the current evidence that epigenetic changes are capable of transmission across generations—so-called “epigenetic inheritance”—when several epigenetic marks are transferred to offspring, who inherit the phenotype in the absence of the external influence [79,80,81,82]. In humans, the transgenerational epigenetic effect has been shown in association with nutrition and food supply. For example, the experience of famine by women in early gestation is associated with glucose intolerance and chronic disease, as well as obesity and cardiovascular diseases, in her children and grandchildren [83,84]. Similarly, there are long-term consequences for the offspring’s later health induced by maternal obesity during pregnancy [85]*) [86,87]. It has even been proposed that epigenetic modifications represent the primary driver or cause of aging (*as a consequence, it has been proposed that an epigenetic assault on aging is a feasible way to reduce multimorbidities in an aging population and even potentially to reprogram the organism to a more youthful state [88,89,90]. The principle possibility of age reprogramming (reverting a differentiated cell back to an induced pluripotent stem cell) was demonstrated by Yamanaka over a decade ago [91]. Since then, reverse programming research has witnessed an explosion [92,93,94,95,96]*) [9,10,34,86,97,98,99,100]. Indeed, older organisms have a different epigenome [101], while individuals with “slower” biological aging have a lower risk for morbidity, disability, and mortality (*for example, it has been shown that each one-year increase in epigenetic age is associated with a 9 percent increase in all-cause mortality, a 10 percent increase in cardiovascular-related mortality, a 7 percent increase in cancer-related mortality, a 20 percent increase in diabetes-related mortality, and a 9 percent increase in chronic lower respiratory disease mortality, even after adjusting for chronological age [102]*) [46,103,104], and in supercentenarians (*supercentenarians are individuals who reach 110-year or longer lifespan [105]*) the epigenetic age is younger than their CA, thus likely playing a significant role in their extremely long lifespan (*curiously, such an association between the epigenetic clock “ticking” and longevity is also observed in other species. For example, the epigenetic clock ticks faster in chimpanzees than in humans [106], which is consistent with the fact that humans have approximately a four-fold greater maximum lifespan than chimpanzees [107]*) [108,109].

However, most epigenetic research in the aging field has largely focused on the relationship of the epigenome with the overall organismal longevity and aging [102,110,111]. At the same time, growing research indicates that such primary causes of death, such as cardiovascular diseases and cancer, are progressively declining [112,113], while mortality due to the fact of neurodegenerative disorders, such as different dementias, Alzheimer’s disease, or Parkinson’s, has increased by 145% over the last 20 years [114,115], thus implying that brain state is an important factor contributing to the overall health- and lifespan (Figure 1). Indeed, cognitive decline, neurodegeneration (*neurodegeneration is one of the most fundamental pathological mechanisms shared by many brain disorders and different subtypes of dementia, including Alzheimer’s disease and Parkinson’s dementia [116]. Neurodegeneration is usually accompanied by impaired neurogenesis [117] and abnormal protein aggregations [118], which are products of dysfunctional autophagy [119], mitochondrial dysfunction, oxidative damage, and inflammation [120,121]*), and many other brain disorders are “champions” of advanced age [28,122], so the brain’s link to the human lifespan is unmistakable, although understudied. All along, the brain contributes to the lifespan directly through a so-called circadian time-keeping system—the “central” circadian clock, which is located in the hypothalamic suprachiasmatic nucleus (SCN) [123,124,125]. This central clock dictates systemic and peripheral circadian behavior and rhythms by synchronizing the neuroendocrine system to the external light–dark cycle [126,127,128]. Disruptions in this central clock result in metabolic deregulation [129], cancer initiation [130,131], and accelerated aging and decreased longevity [132,133,134]. It has been further proposed that the brain also synchronizes the organismal epigenetic clock (including its rate in every tissue—tissue-specific epigenetic clocks [135]), suggesting the central role of the brain in the organismal health- and lifespan [136]. This may explain why an “older” brain may be hostile to a younger body [137] and is also in line with the finding that persons with an older brain age experienced at least two decades of accelerated age-related degradation of the body [138]. Indeed, many neurological and psychiatric diseases (such as schizophrenia, depression, epilepsy, HIV encephalopathy, Alzheimer’s, and traumatic brain injury) are associated with premature or accelerated aging (for an overview, see [139]; see also [140,141]). These observations have recently been supported by the estimation of the epigenetic clock rate: epigenetic aging is accelerated in schizophrenia [142,143], depression [144,145], post-traumatic stress disorder [146], HIV infection [147], Alzheimer’s disease [148], Huntington’s disease [149], and Parkinson’s disease [108].

There is, however, another important “product” of brain activity—subjectivity [150,151,152]—which has largely been ignored until recently in relation to aging and longevity [153,154] but which, nevertheless, stresses the importance of the brain for longevity. Indeed, the subjective perception of age may have profound effects on health and well-being, and it is connected to an individual’s lifespan [153,155]. For example, in a study using 2.253 adults, it was shown that an older subjective age was associated with accelerated epigenetic aging [156]. A link between subjective age and the probability of mortality has been established in three large samples [157]: a subjective age of approximately 8, 11, and 13 years older than CA in the three samples was correlated with an 18%, 29%, and 25% higher risk of mortality, respectively. This link was confirmed in a meta-analysis of 19 longitudinal studies [155]. Recently, Zhavoronkov et al. [154] have shown that a subjective age that is +5 years more than the CA is associated with a more than two-fold increase in the mortality rate, and a subjective age that is –5 years less is clearly a major life protective factor (*these findings have been corroborated by data obtained at the molecular level measuring the length of telomeres [158]. Telomeres are DNA–protein complexes that cap chromosomal ends, promoting chromosomal stability [159], and their length is a factor limiting the maximum number of cell divisions (i.e., the Hayflick limit) and the regenerative potential [160]. Telomeres shorten with age (i.e., the so-called “telomere attrition”) and, thus, telomere length often serves as a biomarker of cellular aging—senescence [161,162]. It was shown that an older subjective age is related to shorter telomeres, beyond what is expected as the CA effect [158]*). Furthermore, a younger subjective age is associated with a lower risk of major depressive episodes [163], while an older perceived age predicts higher depressive symptoms or full depression in the future [164,165]. Additionally, a younger subjective age is associated with improved cognitive functioning 10 years later [166] and is associated with personality traits such as openness, conscientiousness, agreeableness, and extraversion [167] (see also [154]). Interestingly, elderly individuals that reported a subjective age similar to or younger than their actual CA have higher grey matter volume in several brain areas, and this subjective age was a reliable predictor of brain age [168]. Overall, people who feel subjectively younger have more resources, better mental and physical health, higher cognitive abilities, enhanced resilience to stress, a younger biological age (as measured by the epigenetic clock), and a longer lifespan [153,154] (see also [156,169]).

Hence, we argue here that brain aging is the strongest risk factor for health- and lifespan, and it is a major contributor to quality of life and subjective well-being associated with the extension of lifespan and longevity (Figure 1). Thus, establishing effective biomarkers of brain aging is particularly important to better understand the aging process and contribute to a long healthspan by reducing neurodegenerative diseases of aging [170]. Furthermore, such brain age biomarkers may help guide the development of interventions to slow the aging process and extend the healthspan of the whole organism (not just the brain). Indeed, considering that the brain is a “chief” organ (*in fact, contemporary neuroscience increasingly regards the health of the brain as being key to mental and general health, especially in light of new discoveries of the brain’s compensatory properties for the weak function of vital organs of the organism [171]*) which controls, regulates, modifies, or modulates a multitude of physiological (and psychological), neuroendocrine, and immune processes [172,173,174], it contributes to multiple age-related comorbidities [139,175] (*for example, cognitive decline and increased Alzheimer’s disease (AD) risk are associated with coronary heart disease, hypertension, and type 2 diabetes [59]*). Thus, considering the “competing risks argument” [176], one may expect that reducing brain aging could also have a high impact on systemic/organismal life expectancy and healthspan, because the brain rejuvenation effect should be “felt” across multiple tissues and, hence, reflected in many age-related diseases. Indeed, it has been demonstrated recently that overexpressing sirtuins (*sirtuins (SIRT1–7) are a family of nicotinamide adenine dinucleotide (NAD^+^)-dependent deacylases with many roles that prevent multiple diseases (control of energy metabolism, cell survival, DNA repair, tissue regeneration, inflammation, and neuronal signaling) and can even reverse aspects of aging, as well as prolong life [177]*) exclusively in the mouse brain resulted in a longer mean lifespan of the whole organism, as well as a significant increase in the maximal longevity (*importantly, sirtuin levels decline in the brain with age, and this relates to an overall health decline [178]. This process is associated with an age-dependent reduction in NAD^+^ levels in the brain of healthy individuals [179] and also with accelerated brain aging [180]*) [181].

### 1.1. Brain Biological Age Estimation

What could be an appropriate measure of *brain biological age* (BBA)? Currently, there are several biological (epigenetic) “clocks” available that are based on DNA-methylation (DNAm) profiles (*additionally, recent advances in artificial intelligence have allowed the development of other age biomarker measures based on (i) blood biochemistry [44,182], (ii) transcriptomics and proteomics [183], and (iii) the microbiome [184]*); these are (i) the DNAm age clock [185], (ii) the DNAm age H [186], (iii) the DNAm PhenoAge [102], and (iv) the GimAge or DNAm age G [187]. Although it is well known that the aging process exhibits a tissue-specific signature [188,189] and that DNA methylation patterns are distinct between tissue and cell types [190], epigenetic clocks encompass pan-tissue aging changes, and all of them do not perform optimally in human brain tissue (*this does not mean that a meaningful association between systemic DNAm age and neuropathology was not found. On the contrary, there is a robust association between DNAm and Alzheimer’s disease and Parkinson’s disease [191,192]. Moreover, accelerated DNAm age is associated with specific markers (e.g., neuritic plaques, diffuse plaques, and amyloid-b load) of Alzheimer’s disease and declining global cognitive functioning and deficits in episodic and working memory in persons with Alzheimer’s disease [102,193,194]*) [190] (see also [195]), and brain aging also does not correlate with epigenetic aging ([196] and references within). Furthermore, almost all DNAm clock measures are invasive; they require either blood samples or samples derived from certain tissues of the organism, which impose multiple limitations on their usage in experimental settings and real-life applications [29]. As a consequence, these make DNAm clocks unsuitable for routine BBA estimation in living humans. Ideally, the BBA measure should be easily available, cheap, and noninvasive (Figure 1).

Structural brain changes during normal aging comprise progressive decreases in grey and white matter (*grey matter refers to the totality of neuronal cell bodies (also named soma), while white matter denotes the totality of myelinated axons, which are long relays that extend out from the soma (and which are whiteish in color due to the relatively high lipid content of the myelin protein that sheathes them) and form connections between neurons [197]*) [198], which together are a major contributor to morbidity and loss of independence in older adults [199]. For example, postmortem brain studies indicate that myelin lipid loss (part of white matter) is progressive throughout adulthood, exceeding a 40% decrease by 100 years of age [200]. Furthermore, long-distance connections show age-related reductions in both anatomical and functional connectivity [201]. These changes are associated with both general cognitive ability and processing speed decreases [202,203]. However, there is a significant interindividual variability in structural brain aging among older adults [204,205], which is uncoupled from CA, sex, education, or clinical markers such as body mass index (BMI) or uric acid [198,206,207,208]. Indeed, some older individuals experience strong and early manifestations of brain degeneration (i.e., *accelerated brain aging*), while others of comparable age do not experience the brain changes expected at that age (i.e., *decelerated brain aging*) [169,205,209,210]. Magnetic resonance imaging (MRI) of the brain can reliably detect subtle signs of brain *structural* aging decades before the onset of age-related disease [211,212]. These observations led to the emergence of the concept of *brain age,* which is a value estimated using a machine learning algorithm that is trained to predict CA from grey and white matter measures in several independent samples of individuals [53,213,214,215,216]. It was shown that age-related alterations in the brain structure that make the brain appear “older” are associated with Alzheimer’s disease, type 2 diabetes mellitus, a higher BMI, elevated cholesterol and fasting glucose levels, higher diastolic blood pressure, epilepsy, greater smoking and alcohol consumption, more severe depression, and mortality [54,55,141,207,215,217,218]. In summary, MRI-derived brain age reflects only *structural* brain aging—brain atrophy [169] (*additionally, MRI is expensive, nonportable, and usually associated with high stress due to the loud noise and confined space [219]*).

However, a converging line of evidence suggests some level of *decoupling between structure and function* in the brain [220]. Indeed, observations in neurology demonstrated that (a) there is a relative disconnect between the clinical presentation and the underlying neuropathology or amount of brain damage—quite often patients that sustain severe, extensive, and irreversible bilateral physical brain damage have preserved functions or eventually recover in part or fully over time [221,222,223,224,225,226]; (b) different neuropsychological profiles are observed in patients with similar brain damage [223]; (c) in spite of a strong link between physiological and clinical health markers with structural brain aging, often no effects on cognitive scores are found [207]; (d) cognitively unimpaired elderly subjects are characterized by structural changes in the brain that reflect accelerated aging [207]; at the same time, (e) full pathologic criteria for Alzheimer’s disease have been observed postmortem in 25–67% of brains of elderly individuals with no indication of cognitive impairment prior to death [227,228]; and (f) one-sided injury or removal of any given cerebral cortex area does not abolish conscious thinking [229]; moreover, often, higher-order cognition in its core remains generally quite robust, even after extensive and bilateral focal brain damage [220].

Considering all of the above, it seems that a structurally based brain age measure cannot capture the full complexity of the BBA. In this respect, the *quantitative electroencephalogram* (qEEG)-based BBA could be a more suitable, rather simple, and promising measure of brain aging (*an electroencephalogram (EEG) is a summation of the electrical activities along the scalp generated by the firing of nerve cells (i.e., neurons) in the brain [230]. The aggregate of these electric voltage fields creates an electrical reading, which electrodes on the scalp are able to detect and record [231]. qEEG (quantitative EEG) is a digitally recorded and mathematically/algorithmically/statistically analyzed EEG [232]*). This is so because qEEG, in addition to being relatively cheap, portable, nonstressful, and noninvasive, has a number of useful and important characteristics or properties, most of which are age-related or age-dependent (Figure 1):

(a) It constitutes a neural trait measure due to the fact of its high specificity (i.e., the extent to which an qEEG pattern is uniquely associated with a given person) and intra-individual high stability (test–retest reliability) [233,234,235,236,237];
(b) qEEG is highly heritable and, thus, likely to be under strong genetic control [234,238,239,240];
(c) It reflects both the brain’s structural characteristics (or “hardware”) such as the number of connections between neurons, fiber density, axonal diameter, degree of myelination and white matter integrity, as well as the integrity of the corticocortical and thalamocortical circuits, hippocampal volume (*the hippocampus is a brain region central to both healthy memory function and also age-related memory decline [241]*), number of active synapses in thalamic nuclei, brain hemodynamics and metabolism, and the number of potential neural pathways [231,242,243,244] and cognitive processes and functions (“neuropsychological competence” or “software”), such as memory performance, attention and processing speed, individual capacity for information processing (the capacity for storage, transfer, and retrieval of information) and cognitive preparedness (the brain’s capacity for higher-level cognitive functioning), network efficiency, and neural compensation at all ages, both in healthy individuals and in individuals with neurological conditions [245,246,247,248];
(d) qEEG possesses age-related changes in both brain structural and functional integrity (in)dependently of pathology [245,249,250,251,252], thus directly reflecting an aging process;
(e) It shows age-dependent changes that parallel neurological changes in typical aging [253]; indeed, it is known that, for example, atrophic brain regions detected in patients with dementia largely overlap with regions showing normal age-dependent decline in healthy individuals [254];
(f) qEEG is associated with age-related conditions, such as cognitive decline, Alzheimer’s disease, mild cognitive impairment, vascular dementia, other dementias, multiple sclerosis, and cerebral tumors [244,255,256,257].

Capitalizing on these facts, we could conclude that the dualism of the brain’s anatomical (i.e., structural) and cognitive (i.e., functional) reserves can be unified within a single concept—*brain resources* (BR), which can be measured by qEEG. Thus, qEEG-based BBA can be considered a proxy for the BA of the brain. In this context, a person with high BR (brain reserve (“hardware”) + cognitive reserve (“software”)) (*the brain reserve is a “passive” form of capacity that is dependent on the structural properties of the brain, such as a higher number of healthy synapses and neurons [258]. In this context, as brain volume or synaptic density decreases with age, individuals with more premorbid brain reserve will manifest symptoms later in life and less severely than individuals with less premorbid brain reserve—a compression of morbidity that improves quality of life [22]. On the contrary, cognitive reserve describes an “active” function of the brain that involves cognitive operations and representations [258] and refers to the ability to use alternative functions when a default function is rendered inoperable or to the robustness of a particular cognitive function against brain age-related pathologies (see also [259,260]). For example, it has been documented that elderly individuals with a lower cognitive reserve need to over-recruit neuronal networks (due to the lower efficiency and decreased structural properties of their neuronal networks), exhibiting less efficient brain functioning, to achieve the same level of cognitive performance as elderly individuals with a higher cognitive reserve [261]. Moreover, elderly individuals need higher activation of their neuronal networks than young individuals, for the same reason—lower efficiency and decreased structural properties of the elderly subjects’ neuronal networks [262] (see also [263])*) has a younger brain phenotype (qEEG-based BBA) and is more likely to remain within normal (healthy) limits for a longer period of time [209,264]. Conversely, a person with fewer BR has an older brain phenotype (qEEG-based BBA). Indeed, it has been shown that an individual’s brain age can be reliably estimated from qEEG [137,249,250,265,266], and qEEG-derived increased BBA is associated with neurological and psychiatric diseases, diabetes, and hypertension [266], as well as reduced life expectancy and increased mortality risk in comorbidities, such as cardiovascular dysfunction, current smoking status, and increased body mass index [137]. Thus, qEEG-based BBA is a practical, simple, and compelling indication of the BA as opposed to the CA of the brain. It measures the full complexity of brain aging and age-related risks [137,266]. This justifies the use of such qEEG-based BBA to estimate the effectiveness of putative interventions aiming to ameliorate brain aging at a practical (i.e., limited) timescale.

### 1.2. Choosing a Brain Anti-Aging Intervention

The most promising strategy to tackle aging as a whole is by targeting the epigenetic regulators associated with the aging process [34,86,267,268]. The same also applies to brain aging, since identical aging mechanisms are involved, and, as we discussed above, the brain is at the center of organismal processes and functions [172,173]. In this regard, there is growing evidence that the very same interventions that target epigenetic regulators across differently aged tissues have a concomitant anti-aging effect on the brain [170,267,269,270,271,272]. Currently, the most accessible anti-aging interventions that work through epigenetic regulation are physical exercise [90,272,273,274,275,276], diet strategies (for example, caloric restriction and intermittent fasting [90,271,275,276,277,278]) and nutritional supplementation (e.g., vitamins and macro- and micro-elements) [90,268,279,280,281,282,283,284].

Among these strategies, nutraceutical supplements, which are compounds of vitamins, minerals, and essential amino- and fatty acids, as well as plant extract isolates [21,282], may have further advantages (Figure 1): they (i) are widely available and commonly used; (ii) they affect a highly evolutionarily conserved nutrient-sensing pathway (*this pathway regulates several key homeostatic processes, including autophagy, mRNA translation, and metabolism, each of which affects the hallmarks of aging [13,34] and, consequently, the lifespan [285,286]*) linked to aging [287,288]; (iii) could prevent or slow the progression of a wide variety of illnesses [90,283,284], including neurodegeneration [289,290,291]; (iv) can affect the central circadian clock in the brain via sirtuins [134,292], which are linked to the regulation of aging [9,177,293,294]; and (v) do not require as much effort to comply with recommendations, for example, committing to regular physical exercise [295,296,297] or maintaining a rigorous diet [298,299,300,301]. Moreover, considering that many nutraceutical compounds are mimetics of calorie restriction [302] or physical exercise [303], manipulating the dosage of such compounds could achieve stronger and faster results.

As a consequence, it is plausible to hypothesize that an individually tailored (*the strategy of using personalized interventions to meet individual health needs as opposed to a “one-size-fits-all” approach has been recently proposed by Fahy et al. [268] and has shown encouraging results [268] (see also [304]). The need for the personalization of anti-aging interventions has also been recently reiterated [90,305]*) program of nutraceutical compounds may delay or even reverse the BA of the brain, thus increasing the healthspan (the period spent free of chronic disease [306]) and lifespan (the period spent alive [307]) by targeting and manipulating multiple biological pathways that cause aging [34,308]. Furthermore, we expect this approach to be more efficient than lifestyle changes.

### 1.3. Aim of the Study

Therefore, the aim of the present study was to examine whether an individually tailored program of multiple nutraceutical compounds can (a) increase BR (measured by qEEG), thus establishing a younger brain phenotype (younger qEEG-derived BBA), to return the normotonic older brain to a level more comparable to a younger brain (i.e., *rejuvenation*), and/or (b) slowdown the speed of aging of the brain (i.e., *deceleration*) in a cohort of “normal” adults. The lifestyle change group served as an active control.

## 2. Methods

### 2.1. Participants

The participants’ EEG, clinical/medical, and demographic data were extracted for the retrospective analysis from the electronic record registry of BM-Science (*N* = 1.010 on the day of the study onset; the period for the data extraction was between 2013 and 2020). Subjects in this registry (initial cases) during this period were self-selected to receive well-being guidance (other cases in the registry are either participants from previous studies or were referred by doctors for neurophysiologic evaluations). The participants’ data were entered into the study in consecutive order as they met the inclusion criteria until a total of at least 40 individuals in each group (experimental and active control) was obtained in order to have sufficient statistical power (80%) to detect the interventions’ effects. After the inclusion/exclusion criteria were met, the data of 42 (31 females; mean age: 54.1 ± 13 years) and 47 (25 females; mean age: 45.2 ± 7.3 years) participants (for the experimental and control groups, respectively) were included in the analysis. The inclusion criteria were male and female volunteers, aged 25 and above, self-selected to receive either a nutraceutical compounds program (*experimental group*) or lifestyle recommendation (*active control group*), able to follow the intervention for 6 to 18 months, availability of complete pre- and postintervention data, and signed informed consent. The exclusion criteria were: malignancies as suggested by personal medical history, treatment-resistant significant bradycardia (<55 bpm) or hypertension (systolic > 160 mmHg or diastolic > 90 mmHg), allergy/sensitivity to the studied nutraceutical compounds, alcoholism or drug addiction, a diagnosis of schizophrenia, epilepsy, Alzheimer’s disease or Parkinson disease, and no signed informed consent (*the presence of various health complaints and different comorbidities was not qualified as an exclusion criterion for pragmatic reasons so that the study sample was more representative of the general population of “practically” healthy persons, where various health issues are commonly experienced*). The demographic and clinical data, as well as baseline values of BBA and brain resources, are presented in Table 1. This retrospective study can be considered as *single-blind* because the participants were blinded to the interventions’ primary output related to the qEEG-derived BBA (the participants thought that the respected interventions aimed to improve their general well-being).

This study was carried out in accordance with the Code of Ethics of the World Medical Association (Declaration of Helsinki) and the standards established by the Review Board of BM-Science—Brain and Mind Technologies Research Centre. Originally, prior to the EEG scanning and interventions, the experimental procedures were explained, and participants signed an informed consent form. The use of the data for scientific studies was authorized by the written informed consent of the subjects and approval by the Review Board of BM-Science—Brain and Mind Technologies Research Centre.

### 2.2. EEG Recording and Acquisition

Ongoing EEG activity was recorded (using a digital EEG recording system—Mitsar) late in the morning to minimize drowsiness in a quiet and dimly lit room for at least 6 min while subjects were seated on a comfortable half-reclining armchair with their eyes closed. The subjects were asked to have a moderate breakfast and refrain from the consumption of psychoactive drugs (e.g., antidepressants and benzodiazepines) and other psychostimulants (e.g., coffee, tea, and alcohol) at the morning of the recording day. During the EEG recording, the subjects were requested to remain in a standard resting state condition (*the resting-state qEEG manifests the baseline mechanics of self-organization that regulate multiple brain systems, adapting the brain and body to an ever-changing environment [309,310]. Thus, the resting-state qEEG reflects the intrinsic default activity that instantiates the maintenance of information for interpreting, responding to, and predicting environmental (internal and external) demands [247,311,312,313,314]*). In this condition, they had to keep their muscles relaxed without any movements/talking and to stay awake, with their mind freely wandering without systematic goal-oriented mentalization.

The following parameters of the EEG recording were enforced: (i) 19 scalp locations (i.e., O_1_, O_2_, P_3_, P_4_, P_z_, C_3_, C_4_, C_z_, T_3_, T_4_, T_5_, T_6_, F_z_, F_3_, F_4_, F_7_, F_8_, F_p1_, and F_p2_) according to the International 10–20 System of the EEG electrode placement; (ii) 256 Hz sampling rate; (iii) monopolar montage with linked earlobes as a reference electrode; (iv) 0.5–30 Hz bandpass; (v) 50 Hz notch filter ON; (vi) electrooculogram (0.5–70 Hz bandpass); and (vii) impedance below 10 kΩ. Throughout the EEG recording, the experimenter monitored the participant’s state and ongoing EEG traces to assist the subject in maintaining an adequate level of vigilance (i.e., avoiding drowsiness and sleep onset).

Artifact removal was performed by visual inspection of the raw EEG data, augmented by a computerized artifact detection and rejection algorithm (for details, see [315], p. 7). Artifact-free EEG data were subjected to a computerized analysis to estimate the BBA and BR.

### 2.3. Estimation of Cerebral Physiological Age as a Proxy of the Brain’s BA—BBA

Briefly, the qEEG-based BBA was estimated using an established linear regression model that has previously been published and described in detail in [250]. The choice of regression as a method of analysis is defined by the continuous process of brain aging, which manifests itself in the gradual accumulation of age-related effects without clear leaps or stages due to the fact of various aging trajectories of the different functional and structural parameters [316,317,318,319]. The regression analysis resulted in a linear dependence between “age-specific” qEEG changes and CA (for details, see [250]). This linear regression model was used to estimate an individual’s BBA based on the qEEG data and calibrated to current data from the BM-Science registry. In short, the EEG time series were first divided into successive and overlapping 2-sec segments, which were windowed, Fourier transformed, and averaged to produce one power spectrum per recording site. Then, the age-dependent EEG feature based on alpha frequency (7–13 Hz) was extracted and averaged across selected EEG electrodes [249,250].

Since brain aging reflects gradual changes in the structure and function of the brain that occur over time and do not result from disease or other gross accidents, the brain’s aging can match the CA (i.e., normal healthy aging) or it can be delayed (i.e., deceleration—negative values of the BBA), facilitated (i.e., acceleration—positive values of the BBA), or reversed (i.e., rejuvenation) [169,205,209,210]. To capture all these conditions, the qEEG-based BBA was estimated at two time-points: the 1st visit—the baseline acquisition (pre-intervention) and the 2nd visit—the follow-up acquisition (postintervention) after 13 months (on average) of interventions. Comparing the 1st and 2nd visit BBAs, it was possible to evaluate the rate of aging (deceleration or acceleration) and direction (healthy aging vs. rejuvenation) in both groups (experimental and active control).

The difference between the estimated BBA and CA normalized to the CA ranged between 16 and 100 years indicates the individual’s BR (*the low boundary of 16 years was taken, because around this time-point, the maturation of the EEG characteristics (i.e., when the EEG patterns become very similar to the mature waveforms of the adult EEG [320,321]) and most brain areas [322] is completed; these are paralleled by the substitution of organismal growth and maturation with the beginning of biological aging on different levels of the organism [45,323]. A 100-year limit was taken as the potential maximum, which is actually rarely reached by humans*). Values “around 0” indicate that the brain’s resources are in line with those typical for the individual’s CA (i.e., healthy aging); “negative values” indicate fewer brain resources for a given CA—the brain has “overspent” resources characteristic of healthy individuals of an older age—an older brain phenotype; “positive values” indicate more brain resources for a given CA—the brain has preserved resources characteristic of healthy individuals of a younger age—a younger brain phenotype.

### 2.4. Interventions

The *experimental group* used an individually tailored program of nutraceutical compounds for 6–18 months (mean: 13 ± 1.13 months). Individual adjustment of the program was based on the qEEG characteristics that deviated from normative values [324,325,326], prenatal and postnatal data, medical history, personal complaints and existing symptoms, medication used, psychometrics (i.e., scores for depression [327], anxiety [328,329], neuroticism [330]), environmental conditions (stress presence), and life habits (alcohol consumption, smoking, and exercising). A tailored program of nutraceutical compounds with documented mechanistic activity on epigenetic pathways [283,284] included probiotics, vitamins, minerals, polyphenols, and omega-3 fatty acids grouped in sets (to maximize the synergetic effect and minimize the potential opposing effects of the compounds) that were timed throughout the day to align with the circadian rhythm and eating time [331], and the month to also be in keeping with the circannual rhythm—the annual variability of physiological processes [332]. While the exact number of compounds, the frequency of their intake during the day and also per month, as well as the dosages, varied for every participant (based on the criteria described above), the overlapping compounds included vitamin C, vitamin D, vitamin A, vitamin(s) B, omega-3, Mg, Zn, alpha-lipoic acid, CoQ-10, Bifidobacterium, and lactobacillus. All participants were asked to take the supplements on a daily basis in accordance with the program.

The *active control group* used a tailored lifestyle recommendation program over 6–18 months (mean: 13.5 ± 1.10 months), since research also suggests that positive health habits may be able to offset earlier deleterious influences [61,62] and even reverse aging [90,278,333]. Individual adjustment of the lifestyle recommendations was conducted using the same criteria as for the experimental group. Tailored lifestyle interventions included dietary recommendations (plant- and fish-centered; low caloric intake; low carbohydrates; and fasting-mimicking), physical exercise (aerobic: cycling, walking, swimming, and jumping; resistance; sustained isometric nonmaximal voluntary contraction; up to 30 min per day and 3–7 days per week), and sleep of 7–8 h per night. The participants were requested to follow these recommendations daily.

**Table 1:** Demographic and clinical data for the experimental (nutraceuticals) and active control (lifestyle) groups.

Characteristics	Nutraceuticals	Lifestyle	*p*-Value	Test Type
Sample size (N)	42	47	Not applicable	Not applicable
Sex (% of females)	73.8	53.2	0.00204	Chi-square
Chronological age—CA (mean/st.d)	54.1 (13)	45.2 (7.3)	0.00048	Mann–Whitney U test
Brain biological age—BBA (mean/st.d)	46.3 (11)	37.7 (9.8)	0.00042	Mann–Whitney U test
Brain resources—BR (mean %/st.d)	9.89 (20)	8.99 (13)	Not significant	Mann–Whitney U test
Healthy lifestyle habits (% of those who have)	16.7	12.8	Not significant	Chi-square
Current health symptoms (% of those who have)	33.3	40.2	Not significant	Chi-square
Past health problems (% of those who had)	64.3	57.4	Not significant	Chi-square
Relatives with mind/brain disorders (% of those who have)	16.7	23.4	Not significant	Chi-square
Anxiety—Beck ^1^ (mean/st.d)	8.2 (7.1)	7.9 (6.5)	Not significant	Mann–Whitney U test
Anxiety—Ham ^2^ (mean/st.d)	8.7 (6.6)	8.6 (5.5)	Not significant	Mann–Whitney U test
Depression—Beck ^3^ (mean/st.d)	6.2 (6.7)	6.5 (4.8)	Not significant	Mann–Whitney U test
Big-5—neuroticism ^4^ (mean/st.d)	2.8 (0.8)	2.9 (0.7)	Not significant	Mann–Whitney U test
Handedness (% of right-handed)	83.3	87.2	Not significant	Chi-square
Marital status (% of married)	73.8	83	Not significant	Chi-square
Marital status (% of divorced)	9.5	12.7	Not significant	Chi-square
Marital status (% of single)	16.7	4.3	0.002712	Chi-square
Education (% of those who have a PhD)	14.3	10.6	Not significant	Chi-square
Education (% of those who graduated from university or institute)	69	74.4	Not significant	Chi-square
Education (% of those who completed high school (≥11–12 years))	16.7	15	Not significant	Chi-square
Job (% of directors or CEOs)	21.4	17	Not significant	Chi-square
Job (% of senior managers)	38.1	38.3	Not significant	Chi-square
Job (% of junior managers)	35.7	38.3	Not significant	Chi-square
Job (% of students or trainees)	4.8	6.4	Not significant	Chi-square
Number of interests or hobbies (mean/st.d)	4.3 (1.8)	3.6 (1.6)	0.0394	Mann–Whitney U test
Smoking (% of those who smoke)	7.1	2.1	Not significant	Chi-square
Alcohol consumption (1–2 drinks * per week; %)	40.5	40.4	Not significant	Chi-square
Alcohol consumption (3–4 drinks per week; %)	47.6	38.3	Not significant	Chi-square
Alcohol consumption (5–7 drinks per week; %)	7.1	8.5	Not significant	Chi-square
Alcohol consumption (8–10 drinks per week; %)	4.8	12.8	0.04808	Chi-square

^1^ Beck Anxiety Inventory [328]. ^2^ Hamilton Anxiety Rating Scale [329]. ^3^ Beck Depression Inventory [327]. ^4^ Big Five Inventory [330] to assess neuroticism as a personality trait of negative emotionality. * A standard drink is the equivalent of a glass of wine or bottle of beer.

Both interventions (nutraceutical compounds and lifestyle recommendations), as used in the present study, are generally considered safe, even when used for a long time [90]. Adherence to the interventions was verified by phone or email communication with the participants.

We hypothesized that if the tailored program of nutraceutical compounds had a specific advantageous effect on BBA that went beyond the effects of the lifestyle changes, then (a) it should not only slowdown (i.e., deceleration) or reverse (i.e., rejuvenation) the brain’s aging, thus improving the BR, but (b) the magnitude of this effect should also be larger than in the control group that used lifestyle recommendations.

### 2.5. Statistical Analyses

In order to compare the longitudinal changes in the BBA and BR scores between the pre- and postintervention endpoints within the same group, the Wilcoxon signed-rank test was employed. Comparisons between the experimental and control groups were performed using the Mann–Whitney U test and the chi-square test (for demographic characteristics). Additionally, we examined differences in the BBAs with respect to the interventions separately for (i) females and males, as well as for (ii) participants with a baseline (pre-intervention) BBA younger and older than their CA.

The reported *p*-values were not corrected for multiple comparisons because all significant test results were highly correlated, making a Bonferroni correction overly conservative and, thus, inappropriate [334,335].

## 3. Results

### 3.1. Demographic Characteristics

A group comparison of the demographic and psychometric characteristics is shown in Table 1. The experimental (nutraceuticals) and control (lifestyle) groups differed in respect to a number of demographic variables: sex, CA, BBA, marital status—% of singles, number of interests and hobbies, and alcohol consumption—% of those who have 8–10 drinks per week. There was no difference between the two groups for the remaining (majority) characteristics (Table 1). Despite the fact that CA and BBA differed between the groups, the BR was nearly identical—this is important for the purpose of the present study, since the qEEG-derived BR score, which is a proxy for the brain’s neurophenomenological condition, was on average identical at the baseline (pre-intervention) time-point, thus ensuring an equal starting point for the participants in both groups (Table 1).

### 3.2. Neurophysiological Findings: BBA and BR

**Figure 2:**
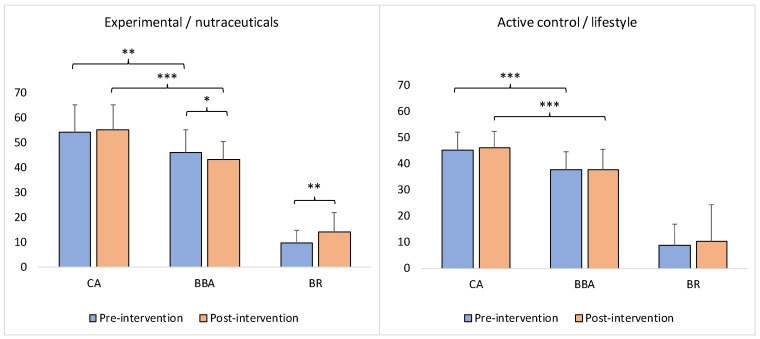
Intervention-induced changes in BBA and BR in the experimental (nutraceutical compounds) and active control (lifestyle) groups. The *y*-axis represents years for the BBA and CA and the percentage for the BR. CA: chronological age; BBA: biological brain age; BR: brain resources. The asterisk(s) denotes *p* < 0.05 (*), *p* < 0.01 (**), and *p* < 0.001 (***). The bars represent the means with standard errors.

The findings of this study show that although, on average, the BBA was significantly younger than the CA at baseline (pre-intervention) for both groups (Wilcoxon signed-rank test: *z* = −2.72, *p* = 0.00652 for the nutraceuticals group; *z* = −3.98, *p* = 0.00006 for the lifestyle group), and both groups had increased BR (+9.89% for the nutraceuticals group; +8.99% for the lifestyle group); the BBA nevertheless significantly decreased and BR significantly increased (+14.16%) as a result of the intervention (post-endpoint) only in the experimental/nutraceuticals group (Wilcoxon signed-rank test: *z* = −2.27, *p* = 0.0232 for BBA; *z* = −3.15, *p* = 0.00164 for BR) (Figure 2). On the contrary, in the control/lifestyle group, BBA and BR did not show a significant change postintervention (Wilcoxon signed-rank test: *z* = −0.42, *p* = 0.67448 for BBA; *z* = −1.48, *p* = 0.13622 for BR) (Figure 2).

At the same time, on average, the BBA continued to be significantly younger in comparison with the CA at the postintervention endpoint of both groups (Wilcoxon signed-rank test: *z* = −4.12, *p* = 0.00001 for the nutraceuticals group; *z* = −4.07, *p* = 0.00001 for the lifestyle group) (Figure 2).

**Figure 3:**
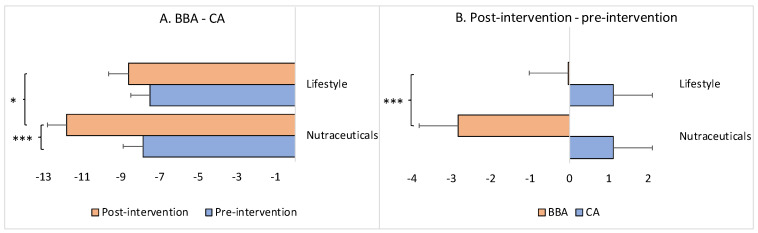
Difference between BBA and CA, as well as between post- and pre-intervention time-points, as a function of the intervention (i.e., nutraceutical supplementation versus lifestyle changes). The *x*-axis represents the difference in years. CA: chronological age; BBA: biological brain age. The asterisk(s) denotes *p* < 0.05 (*) and *p* < 0.001 (***). The bars represent the means with standard errors. (**A**) The negative values indicate that the BBA was younger than the CA (i.e., deceleration of brain aging), a “zero” value indicates that the BBA coincided with the CA (i.e., normal healthy aging), and positive values indicate that the BBA was older than the CA (i.e., acceleration of brain aging). (**B**) The negative values indicate brain age reversal, a “zero” value indicates brain age stabilization (i.e., slowdown), and positive values indicate brain aging.

On average, the decrease in the BBA in comparison to the CA (=BBA-CA) was −7.86 years for the experimental/nutraceuticals group and −7.49 years for the control/lifestyle group at the pre-intervention point and −11.8 years and −8.62 years, respectively, postintervention (Figure 3A). While there was no statistically significant difference between these values for the two groups at the pre-intervention point (Mann–Whitney U test: *z* = 0.36, *p* = 0.71884), postintervention the groups did differ significantly (Mann–Whitney U test: *z* = 1.91, *p* = 0.04961) due to the significant widening of the difference between BBA and CA in the experimental/nutraceuticals group (Wilcoxon signed-tank test: *z* = −3.43, *p* = 0.0006), and no significant difference between BBA and CA in the control/lifestyle group (Wilcoxon signed-rank test: *z* = −1.67, *p* = 0.09492) (Figure 3A).

Furthermore, the BBA of the participants in the experimental/nutraceuticals group was, on average, 2.83 years younger at the endpoint of the intervention compared to the same individuals at the beginning. The BBA of the control/lifestyle participants was, on average, only 0.02 years younger compared to the baseline at the end of the intervention; this difference between the groups was statistically significant (Mann–Whitney U test: *z* = −3.98, *p* = 0.00006) (Figure 3B). As expected, the average CA values in both groups increased as a function of the follow-up time: approximately +1.1 years for both groups, without a statistical difference between the groups (Mann–Whitney U test: *z* = −0.89, *p* = 0.36812) (Figure 3B).

**Figure 4:**
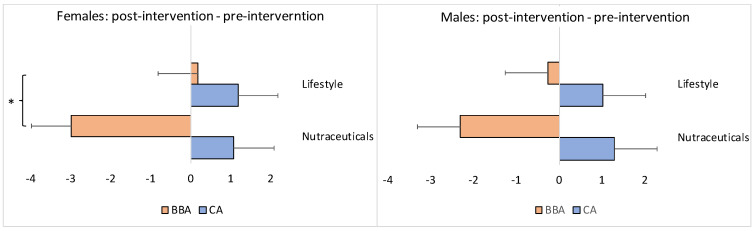
Difference between the post- and pre-intervention endpoints as a function of the intervention (i.e., nutraceutical supplementation versus lifestyle changes) separately for the female and male subgroups. The *x*-axis represents the difference in years. CA: chronological age; BBA: biological brain age. The asterisk denotes *p* < 0.05 (*). The bars represent the means with standard errors. The negative values indicate brain age reversal, a “zero” value indicates brain age stabilization (i.e., slowdown), and positive values indicate brain aging.

Because the results above represent the average values for all participants in each group, they may not accurately capture the impact of the interventions on the different sexes or those whose BBA was either older or younger than their CA at the baseline (pre-intervention) point. Thus, sex, as well as baseline BBA, may be potential confounding covariates of the overall results. Therefore, we conducted separate stratification analyses based on “sex” and the “BBA pre-intervention score”. The stratification analyses revealed the following results (Figure 4).

The BBA of females in the experimental/nutraceuticals group (*N* = 31) scored, on average, 2.98 years younger at the endpoint of intervention compared to the baseline. The BBA of females in the control/lifestyle group (*N* = 25) scored, on average, 0.19 years older at the end of the intervention compared with the baseline; this difference between the groups was statistically significant (Mann–Whitney U test: *z* = −2.02, *p* = 0.04338) (Figure 4). The CA became older in both groups at the endpoint of the interventions: on average, +1.08 years for the experimental/nutraceuticals group and +1.19 years for the control/lifestyle group, without a statistical difference between the groups (Mann–Whitney U test: *z* = 0.35, *p* = 0.72634) (Figure 4).

For the male participants, the results were slightly different. The BBA of males in the experimental/nutraceuticals group (*N* = 10) was, on average, 2.31 years younger at the endpoint of the intervention compared to the baseline. The BBA of males in the control/lifestyle group (*N* = 22) was, on average, 0.26 years younger at the end of the intervention when compared with the baseline; this difference between the groups, however, did not reach statistical significance (Mann–Whitney U test: *z* = 0.42, *p* = 0.6672) (Figure 4). The CA became older in both groups at the endpoint of the interventions: on average, +1.28 years for the experimental/nutraceuticals group and +1.02 years for the control/lifestyle group, without a statistical difference between the groups (Mann–Whitney U test: *z* = −1.12, *p* = 0.26272) (Figure 4).

**Figure 5:**
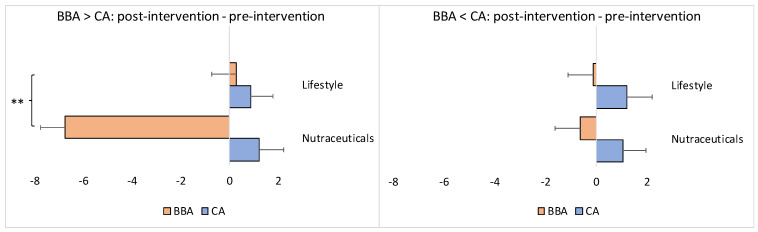
Difference between the post- and pre-intervention endpoints as a function of the intervention (i.e., nutraceutical supplementation versus lifestyle changes) separately for situations when the pre-intervention BBA was older or younger than the CA. The *x*-axis represents the difference in years. CA: chronological age; BBA: biological brain age. The asterisks denote *p* < 0.01 (**). The bars represent the means with standard errors. The negative values indicate brain age reversal, a “zero” value indicates brain age stabilization (i.e., slowdown), and positive values indicate brain aging.

For the participants whose pre-intervention BBA was older than their CA, the BBA in the experimental/nutraceuticals group (*N* = 15) was, on average, 6.77 years younger at the endpoint of the intervention compared to the baseline. For the control/lifestyle group (*N* = 13), the BBA was, on average. 0.25 years older at the end of the intervention when compared with the baseline; this difference between the groups was statistically significant (Mann–Whitney U test: *z* = −2.83, *p* = 0.00466) (Figure 5). The CA became older in both groups at the endpoint of the interventions: on average, +1.22 years for the experimental/nutraceuticals group and +0.86 years for the control/lifestyle group, without a statistical difference between the groups (Mann–Whitney U test: *z* = 1.05, *p* = 0.28914) (Figure 5).

For the participants whose pre-intervention BBA was younger than their CA, the BBA in the experimental/nutraceuticals group (*N* = 27) was, on average, 0.64 years younger at the endpoint of the intervention compared to the baseline. For the control/lifestyle group (*N* = 34), the BBA was, on average, 0.13 years younger at the end of the intervention when compared with the baseline; this difference between the groups, however, did not reach statistical significance (Mann–Whitney U Test: *z* = −0.43, *p* = 0.65994) (Figure 5). The CA became older in both groups at the endpoint of the interventions: on average, +1.04 years for the experimental/nutraceuticals group and +1.21 years for the control/lifestyle group, without a statistical difference between the groups (Mann–Whitney U Test: *z* = −0.37, *p* = 0.70394) (Figure 5).

In order to analyze the potential factors that may be associated with the pre-intervention BBA, we pooled together the demographic and clinical data from both groups and then stratified all participants into two subgroups: BBA > CA and BBA < CA at baseline. The result is presented in Table 2. Some differences between subgroups were expected because they themselves were the basis of the stratification (BBA and related to it BR), while in others they arose originally. The BBA < CA subgroup was characterized by a statistically significant smaller number of right-handed, single, and smoking participants with a total education of high school and a statistically significant higher number of participants who were married, had a PhD, had more hobbies and interests, and consumed more alcohol per week when compared to the BBA > CA subgroup (Table 2).

Since the duration of the interventions varied between 6 and 18 months, it was interesting to see if the changes in the BBA scores were associated with the duration of the interventions. The correlation analysis did not reveal a significant correlation for either group: experimental/nutraceuticals: *r* = 0.25, *p* = 0.110319 (Pearson correlation test); control/lifestyle: *r* = 0.21, *p* = 0.156549 (Pearson correlation test).

### 3.3. Psychometrics and Health Symptoms

**Figure 6:**
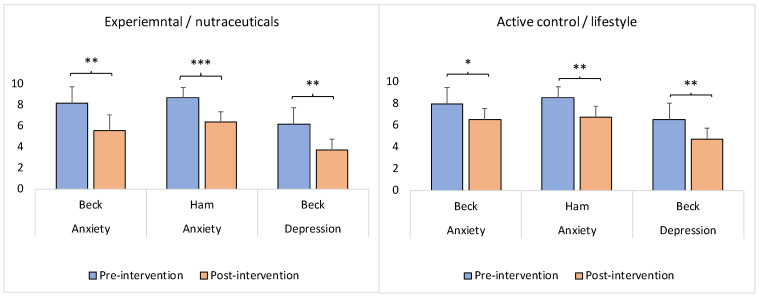
Intervention-induced changes in the anxiety and depression scores in the experimental (nutraceutical compounds) and active control (lifestyle) groups. The *y*-axis represents the standardized scores. Beck: Beck Anxiety Inventory [328] or Beck Depression Inventory [327]; Ham: Hamilton Anxiety Rating Scale [329]. The asterisk(s) denotes *p* < 0.05 (*), *p* < 0.01 (**), and *p* < 0.001 (***). The bars represent the means with standard errors.

While, on average, the experimental/nutraceuticals and control/lifestyle groups did not differ significantly in the scores for depression, anxiety, and neuroticism at the pre-intervention time-point (see Table 1), the postintervention scores for depression and anxiety decreased significantly in both groups as a function of the intervention (Figure 6; experimental/nutraceuticals—Wilcoxon signed-rank test: *z* = −3.19, *p* = 0.00138 (anxiety—Beck); Wilcoxon signed-rank test: *z* = −4.29, *p* = 0.00001 (anxiety—Ham); Wilcoxon signed-rank test: *z* = −2.92, *p* = 0.0035 (depression—Beck). Control/lifestyle—Wilcoxon signed-rank test: *z* = −2.13, *p* = 0.03318 (anxiety—Beck); Wilcoxon signed-rank test: *z* = −2.68, *p* = 0.00736 (anxiety—Ham); Wilcoxon signed-rank test: *z* = −2.95, *p* = 0.00318 (depression—Beck)). Compared to the control/lifestyle group, the magnitude of the significance was larger in the experimental/nutraceuticals group for the anxiety scores measured by both the Beck and Ham tests (Figure 6). At the same time, the postintervention scores for depression and anxiety did not differ significantly between the groups (anxiety—Beck: Mann–Whitney U test: *z* = 0.83, *p* = 0.4009; anxiety—Ham: Mann–Whitney U test: *z* = 0.83, *p* = 0.4009; depression—Beck: Mann–Whitney U test: *z* = 1.38, *p* = 0.1645).

The estimation of the current health symptoms (Table 3) revealed a comparable percentage of participants who experienced them in both groups at the pre-intervention time-point (Chi-square statistic = 1.0571, *p* = 0.303887). However, postintervention, only the experimental/nutraceuticals group had a significant decrease in the percentage of the participants who experienced current health symptoms when compared with the baseline (Chi-square statistic = 25.4711, *p* = 0.00001). In the control/lifestyle group, the decrease was small and nonsignificant (Table 2; Chi-square statistic = 1.3889, *p* = 0.238593).

## 4. Discussion

The goal of the present study was to demonstrate the slowing down or even reversal of the brain BA by means of safe and accessible interventions (nutraceutical supplementation vs. lifestyle changes) in order to ameliorate brain aging at a practical (limited) timescale (Figure 1). The obtained results, while limited, support the feasibility of this goal and also provide the first robust evidence that the regression of brain aging is indeed possible in humans. Compared to lifestyle changes, the intervention involving nutraceutical supplementation was efficient in significantly reducing (i.e., reversing) BBA and enhancing BR at the end of the 13-month (on average, the minimum was 6 months and the maximum was 18 months) program (Figure 2). In contrast, the lifestyle intervention was able to only slow down the BBA and stabilize the BR, keeping them at the same rate as before the intervention (Figure 2) despite the increase in CA. The BBA was 11.8 years younger than the CA in the nutraceuticals group at the end of the intervention (*such a difference between biological and chronological ages is comparable with the differences reported in previous studies: 12 years [336], 12.6 years for Hannum’s epiclock, and 17.5 years for Levine’s epiclock [268]; 15.3 years for females and 16.7 years for males [39]*). This difference was significantly larger than at the beginning of the intervention (Figure 3A). For the lifestyle group, the BBA was 8.62 years younger than the CA at the end of the study, although this difference was not significantly different from the beginning of the study (Figure 3A). The BBA of the participants in the nutraceuticals group was 2.83 years younger at the endpoint of the intervention compared with the baseline BBA (*again, such a rate of reversal in the BA is comparable with reported rates in previous studies: 2.5 years [268] and 1.96 years [90]*), while the BBA of the lifestyle participants was essentially unchanged, measuring only a few days younger compared to the baseline (Figure 3B).

Together, these findings provide substantial evidence that nutraceutical compounds (vitamins, minerals, and essential amino- and fatty acids, as well as plant extract isolates, such as polyphenols [21,282])—when used in specific combinations and adjusted individually—may reverse BBA and increase BR. While the exact mechanisms involved are not clear, one may speculate that different nutraceutical compounds probably have unique and often small effects that are in opposition to brain aging, and when combined in an individually adjusted fashion, these compounds activate a broad enough range of synergistically interacting metabolic pathways that then restore brain resources and reverse brain biological aging. This suggestion is consistent with the known ability of nutraceuticals to affect a highly evolutionarily conserved nutrient-sensing pathway linked to aging [287,288,337] and lifespan [285,286]; prevent or slow the progression of a wide variety of illnesses [90,283,284], including neurodegeneration [289,290,291,337]; improve cerebral blood flow and antioxidant capacity [338,339]; and, additionally, affect the central circadian clock in the brain via sirtuins [134,292], which are also linked to the regulation of aging [9,177,293,294]. In this regard, as has been proposed by Nur et al. [284], nutraceuticals could even be considered “epidrugs”. Indeed, for example, in addition to its role as a cellular antioxidant [339], vitamin C is a critical epigenome remodeler that ameliorates epigenome dysregulation (by enhancing the activity of Jumonji-C domain-containing histone demethylases (JHDMs) and ten-eleven translocation (TET), which drive histone and DNA demethylation) and restores the youthful state of cells (*additionally, vitamin C can also target α-ketoglutarate-dependent dioxygenases (α-KGDDs), which are essential in regulating metabolism, DNA repair, and DNA/RNA demethylation and plays an important role in fine-tuning the reprogramming stages of youthful states of cells [340]*) [341]. TETs are highly expressed in the brain [342,343], with TET1 and TET3 involved in proper brain and cognitive function [103,344,345], while TET2 is associated with neurogenic processes by restoring adult neurogenesis to youthful levels and, thus, enhancing cognitive function [267] (*neurogenesis is a process of generating new functional neurons in the brain [346]. For a long time, it was thought that the loss of neurons was irreversible in the adult brain because dying neurons cannot be replaced; however, later it was demonstrated that life-long continuous neurogenesis takes place in almost all mammals, including humans [347]*). Vitamin A works synergistically with vitamin C by stimulating TET expression [280] (*for the role of other vitamins in epigenetic modification, see Nur et al. [284], and for the effects of vitamins, polyphenols, and minerals on the cells’ homeostasis, senescence, telomere length, and counteraction of DNA damage, see Proshkina et al. [283]*). Another vitamin (vitamin D) may stimulate the production of neurotrophic, antioxidative, and anti-inflammatory factors; reduce risk of cerebrovascular (as well as cardiovascular) diseases; and even influence amyloid phagocytosis and clearance (*it is known that the aging brain is vulnerable to inflammation, where the circulating proinflammatory factors can promote cognitive decline and are responsible for the loss of macrophages’ and microglia’s ability to clear misfolded proteins in the brain, which are associated with neurodegeneration, dementia, and Alzheimer’s disease [348]*) [349]. Furthermore, a high level of vitamin D is associated with the reduced degeneration of major brain white matter tracts, even in cognitively healthy elderly individuals [349]. Additionally, vitamin D happens to upregulate αKlotho (KL) transcription [350]. KL is a protein that is mainly expressed in the brain and also the kidneys [351]; it has strong anti-inflammatory and neuroprotective properties, making this protein a key factor for health and longevity [78]. Interestingly, some polyphenols have a synergetic effect, making it easier for vitamin D to upregulate KL gene expression [352]. Furthermore, the mammalian target of the rapamycin (mTOR) pathway, which detects high amino acid concentrations, is one of the hallmarks of aging [34]. Its overactivation promotes aging and decreases lifespan (for a review, see [353]), while its suppression is associated with an increase in lifespan (*importantly, lifespan extension is comparable if the anti-aging intervention is initiated at a young age, middle age, or in late life [354]*) [355]. In the brain, upregulated mTOR signaling has been associated with amyloid accumulation and, conversely, downregulated mTOR signaling is associated with reduced amyloid levels [356]. In addition, higher levels of mTOR activation—alongside its downstream effectors—were found in brain regions that were affected by Alzheimer’s disease or mild cognitive impairment [357,358]. Therefore, the inhibition of mTOR is desirable. A number of nutraceutical compounds can do this: vitamin D [359], curcumin [360], EGCG—green tea component [361], omega-3 [362], and alpha-lipoic acid [363]. Another important regulator of aging is adenosine monophosphate-activated protein kinase (AMPK), the increased activity of which is related to an extended lifespan [364]. Studies indicate that the responsiveness of AMPK signaling steadily declines with age [365,366]. AMPK activation in the brain is responsible for neuroprotection through the induction of autophagy, angiogenesis, and neurogenesis [337,367]. It has been demonstrated that some polyphenols with antioxidant and anti-inflammatory properties [368] can activate silent information regulator 1 (SIRT1), which belongs to the Sirtuin family [369] and the activation of which can stimulate the activation of AMPK (*interestingly, AMPK activation may restimulate the functional activity of SIRT1 [370], thus resulting in a positive feedback loop between SIRT1 and AMPK, which, in turn, can potentiate the function of the other AMPK-activated signaling pathways important for healthspan in general [364] and the brain in particular [78]*) [371], thus providing anti-aging effects in the brain (*polyphenols such as resveratrol easily cross the blood–brain barrier (BBB) to express their effects in the brain [372,373,374]*) [78,375,376]. SIRT1 also has another path to affect brain aging: regulation of the central circadian clock [292,377]. Apparently, the loss of SIRT1 in the brain not only dysregulates the circadian clock but also accelerates the aging process [134,294] (*such acceleration is most likely mediated by NAD^+^ [134]. Indeed, an age-dependent reduction in the levels of NAD^+^ in the brain was reported in healthy individuals [179], as well as in accelerated brain aging [180]. Furthermore, considering that the circadian clock regulates the oscillatory dynamics of NAD^+^ levels [378] and that this clock is dysregulated in the aging brain [377], a decline in NAD^+^ levels over a person’s lifespan may be attributed to the loss of circadian clock function [134]. A deficiency in NAD^+^ can be restored by vitamin B3 (and its derivatives) supplementation [379]*). On the contrary, the upregulation of SIRT1 in the brain results in an increase in lifespan [181]. Moreover, the antioxidant carotenoid astaxanthin, especially when combined with folic acid, selenium, zinc, and omega-3, can reduce the degree of hypermethylation [282], which normally shows a robust and progressive rise during CA in the brain [380], as well as in the organism as a whole [9], and it is accelerated in neurodegeneration [148]. Additionally, zinc contributes to genomic stability [381], which tends to destabilize with age [9], and together with selenium, it might prevent or delay Alzheimer’s disease in the elderly with mild cognitive impairment [382]. Higher omega-3 levels are associated with greater total grey matter, total brain volume, and lower white matter lesion volume [383]. Omega-3 has been shown to display a decreased concentration in patients with dementia or predementia syndrome [384], while supplementation with omega-3 improved cognitive function in elderly patients with mild cognitive impairment [385] and Alzheimer’s disease [386]. Taking these observations together, one may conclude that there are multiple ways in which an individually tailored combination of nutraceutical compounds may contribute to BBA reversal, as well as BR enhancement, by modulating the epigenome [280], thus safeguarding physical and mental health during CA, and hypothetically even reducing mortality [281].

In contrast to the nutraceutical supplementation intervention, the lifestyle change intervention was quite effective in slowing down the brain BA and maintaining BR, thus stabilizing them against the natural and inevitable pressure of CA (Figure 2 and Figure 3). This is in agreement with previous research that suggests the beneficial effects of healthy habits over life [61,62]. Indeed, a healthy lifestyle that incorporates regular physical activity and a balanced diet promotes multiple anti-aging processes in the organism and the brain [205] and may even reverse the epigenetic age [90]. For example, the beneficial effects of physical exercise (through a mediation of glycosylphosphatidylinositol-specific phospholipase D1, which increases after exercise) on neurogenesis in the aged brain and to improve cognition have been recently demonstrated [272]. Neurogenesis progressively declines with age [387]; its decline is exacerbated in Alzheimer’s disease [388], correlates with cognitive dysfunction [389], and contributes to lifespan duration [390]. Thus, maintaining higher levels of brain neurogenesis is proposed to be neuroprotective and responsible for a rejuvenating/regenerative capacity in the aging brain [387], as it is linked to enhanced cognition and slower disease progression in the context of Alzheimer’s disease [388]. Generally, regular physical exercise plays an essential role in maintaining healthy neurocognitive function (especially in chronologically older individuals) [391], preservation of brain grey matter [392] and hippocampus volume [347], upregulation of neurotrophic factors, including brain-derived neurotrophic factors [393], and maintaining a healthy central nervous system immunometabolism during aging [394]. Similarly, a calorie restriction diet has been systematically demonstrated to extend both the life- and healthspan and to delay many aspects of aging (*for example, the well-documented good health and high number of centenarians among the population of the Japanese of Okinawa island have been attributed to calorie restriction [395]*) [396,397,398]. When it comes to the brain, diet, and specifically a fasting-mimicking diet, has been shown to be able to enhance remyelination (*myelination refers to the process of creating myelin on the neuron axons (the nervous system’s “wires”), whereas myelin is a lipid-rich (fatty) substance that surrounds axons to insulate them and increase the rate at which electrical impulses (called action potentials) are passed along the axon [399]. In the central nervous system, axons carry electrical signals from one nerve cell body to another*) in the aging brain by affecting the oligodendrocyte precursor cells [271] (*oligodendrocyte precursor cells (OPCs) differentiate into mature oligodendrocytes, which myelinate axons in the mammalian brain, allowing for the rapid propagation of action potentials and metabolic support of axons [271]. While most myelination occurs during early postnatal development, OPCs persist in the adult brain [400]*). The deeper mechanism at play is that the fasting-mimicking diet upregulates AMPK activity, which, in turn, inhibits mTOR activity in the oligodendrocyte precursor cells, leading to a markedly increased differentiation capacity of such cells, reminiscent of the young brain [400]. Furthermore, a fast-mimicking diet also leads to SIRT1 activation [369] and increased expression of mesencephalic astrocyte-derived neurotrophic factor (MANF) (*MANF is an evolutionarily conserved protein [401] that is expressed by most tissues in the body [402] and is cytoprotective in multiple systems [270]*) in the brain [403]. It is known that MANF levels progressively and significantly decline with age; however, its overexpression prevents age-related inflammation, deregulates metabolic function, and results in significant mean and maximum lifespan extension in animal models [404]. Thus, existing evidence highlights the benefits of lifestyle management as an effective intervention capable of slowing down brain aging. However, one has to follow such a rigorous program rather precisely on an everyday basis to achieve results beyond just the deceleration of aging, namely, the reversal of brain age, which is not easy in real life, where slowly accruing benefits may not be reaped or noticed (especially in the healthy/young) for decades to come. The difficulty of long-term compliance (which is well documented for the lifestyle changes [296,297,300,302]) was probably responsible for the fact that in our study only a slowdown (i.e., deceleration) of brain BA was achieved with the lifestyle intervention and not actual brain age reversal (*this is in contrast to a much shorter (eight-week) lifestyle intervention (that included diet recommendations, physical exercise, and sleep advice) study, where the systemic/organismal BA was reversed by the end of the study [90]. The duration of the trial may, in fact, contribute to this discrepancy, because it might be easier for participants to follow the intervention program accurately for a much shorter time (by comparison, our study’s intervention duration was, on average, 13 months). Furthermore, while the study by Fitzgerald et al. [90] did not involve any nutraceutical compounds, it nevertheless allowed participants to continue using some nutraceuticals that they had used before enrolling in the study, thus creating a synergistic effect, where nutraceuticals worked alongside the lifestyle recommendations. Furthermore, our study estimated the qEEG-derived brain BA, while the study by Fitzgerald et al. [90] measured epigenetic systemic BA, which may have contributed to the difference in the results*). Moreover, many nutraceutical compounds are, in fact, exercise or calorie restriction mimetics [302,303] (*mimetics are compounds that activate (mimic) the same metabolic, biochemical, and physiological response pathways induced by calorie restriction (or fasting) or physical exercise without lowering food intake or practicing exercise [405,406]*). Thus, with a proper dosing regimen and using combinations that reinforce the effects of separate compounds, one could amplify the beneficial effects of physical activity and diet and, thus, achieve stronger effects. This may explain why not only brain age deceleration but also brain BA reversal and an increase in BR were achieved with nutraceutical supplementation in the present study.

Despite this difference (brain BA deceleration for the lifestyle intervention vs. BA reversal for the nutraceutical supplementation intervention), both results of the studied interventions are, in fact, important, as one may expect that each chronologically passing year (CA) produces less damage and smaller deteriorations in brain health (BA), thus resulting in a slower brain aging and, as a consequence, a greater gap between the biological and chronological age of the brain (Figure 3A). However, the intervention involving nutraceutical supplementation had an additional advantage: the BBA reversal was also accompanied by a dramatic decrease in the number of individuals who had ongoing health complaints (Table 3). This result is significant, especially in light of the current understanding that interventions that target aging have a greater impact on life expectancy and healthspan when the incidence of multiple diseases is reduced—compressed morbidity [12] (see also [16,25,176]). The analogous decrease in the lifestyle intervention was small and nonsignificant (Table 3). At the same time, our results show that both interventions effectively and similarly decreased the scores for depression and anxiety (Figure 6), thus having a comparable effect on mental health. Considering the known correlation between mental health and subjective age [153], we hypothesize that both interventions resulted in a decreased subjective age (*importantly, it has been shown that subjective feeling regarding personal age is associated with brain BA [169]: persons who had an older brain BA reported that they felt less healthy and older than their CA; additionally, they also reported that they looked older than their CA and did not feel likely to live past 75 years. Such individuals had a thinner and smaller cortex, reduced hippocampal volume, and displayed early signs of white matter deterioration, as well as cognitive decline [169]*). Since personal attitude towards aging is strongly associated with the incidence of age-related diseases, epigenetic aging, and mortality [154,156], modifying it by means of such interventions could be a simple and accessible way to increase human healthspan and improve well-being.

One may consider that the reported BBA decrease of 2.8 years (after nutraceutical supplementation for approximately 1 year) is rather modest; however, such a decrease, if sustained, is likely to have a significant impact on personal health risks and well-being, as well as broad economic and societal benefits [8,12,407,408]. Indeed, it has been documented that slowed brain aging is associated with an increase in compensatory and neuroprotective mechanisms and an increased ability to maintain focus, adapt flexibly and quickly to new circumstances, integrate across multiple sensory modalities, and learn efficiently, while accelerated brain aging is associated with an increased risk of Alzheimer’s disease and other diseases that are typically accompanied by cognitive decline, as well as increased mortality [54,55]. Furthermore, the postintervention difference between brain BA and CA was very large in our study—BBA was 11.8 years younger than CA. In and of itself, this is remarkably significant; for example, for the organismal BA, it has been shown that for every 1-year increase in the calculated difference between the BA and CA (when the BA was older than the CA), the hazard ratio for mortality significantly increased by 1.6% (1.5% in males and 2.0% in females), as well as for hypertension (2.5%), diabetes mellitus (4.2%), heart disease (1.3%), stroke (1.6%), and cancer incidence (0.4%) [47]. So, a younger age is associated with better prognoses for a variety of leading sources of human mortality, including, of course, the ongoing SARS-CoV-2 pandemic [409,410,411]. All these have relatively straightforward benefits to individual health- and lifespan; however, where society, as a collection of many individuals, is concerned, the economic benefits begin to emerge as well [21]. While some see health- and lifespan extension as a problem for society (for a review, see [412]), others show that there are, in fact, serious overall economic and societal gains to be had. It has been calculated that a slowdown in aging that increases life expectancy by one year is worth USD 38 trillion, and an increase of ten years is worth USD 367 trillion [12] (see also [407,408]). This is because biologically younger brains correlate with a longer life- and healthspan [178,181], where more people are alive at older (chronological) ages in better health (biological age), thus compressing morbidity [16,21,22,25]. So, when reaching older ages in good health, individuals also tend to (re)allocate more consumption, leisure, and productivity to these years, as they become more valuable [12]—people want to live long but with an ever-stronger interest in remaining healthy and living well [21]. In the words of Scott and colleagues [12], this situation creates a virtuous circle, such that the more successful a society is at improving how people age, the greater the economic and also individual value of further age improvements (*however, not everyone is so optimistic. For example, Davis [413] asked if radical life extension would have value, meaning that such a life would have the unity or coherence to be recognizably human or whether a very long life must invariably become tedious. He also raises moral and political issues, for example, fairness, by asking who would be able to afford the life-extension interventions and whether such interventions would be accessible to everyone*).

Moreover, in our study we did not find any association between BBA and the duration of the interventions. One explanation could be that the beneficial effects of the interventions on brain BA (either its reversal by nutraceutical compounds or its stabilization by lifestyle) were effectively achieved during the first 6 months and then remained relatively stable. This interpretation is consistent with mathematical projections from a large-scale study, according to which the effects of a given longevity intervention in a “practically” healthy population will saturate in a relatively short period of time [414], but somehow this is in contrast with the observation of the systemic BA (estimated by four different epigenetic “clocks”), where there was a marked acceleration of BA reversal after 9 months of intervention that included recombinant human growth hormone, dehydroepiandrosterone, and metformin [268]. This discrepancy remains to be explored; however, it might be that hormonal and medication usage require more time to “kickoff”, or it could be that the brain is a faster responder than the whole organism.

Another compelling result of the present study was that the baseline (pre-intervention) BBA was generally younger than the CA in both groups (Figure 2). This result is consistent with the estimation of organismal BA, which has repeatedly been shown to be lower than CA [111,190,195,268,415,416]. The same dependency was also found for psychological age, where people have a tendency to perceive themselves as younger than their calendar age [417,418], and curiously, this difference increases with CA [419,420]. Taken together, these observations (including the present study) may signify the existence of some deep mechanism that keeps BA and BBA systematically younger than the CA in the human population, thus uncoupling aging from the fixed progression of chronological time [74]. This may explain why humans are generally rather resilient [220] and the longest living among their closest ape “relatives” [107]. It must be noted, though, that this result reflects the average for the groups and that both groups had participants whose BBA at the pre-intervention time-point was either younger or, on the contrary, older than their CA. To analyze these participants, we pooled together data from both groups and then stratified the whole sample into two subgroups: pre-intervention BBA > CA and BBA < CA (see Table 2). The demographic data revealed that the participants whose pre-intervention BBA was younger than their CA had more BR, were more likely to be left-handed, were predominantly married, were more likely to have a PhD, enjoyed more hobbies, smoked less, and consumed more alcoholic drinks per week (Table 2). Largely, these findings are consistent with previous observations: the degree of education, marriage and socialization, diverse leisure activity/hobbies, and increased cognitive reserve were all associated with higher cognitive performance, neuroprotection, and resilience to neurodegeneration and Alzheimer’s disease [57,205,421,422,423], as well as with younger systemic (organismic) epigenetic age [102]. For example, it was found that superagers (or “high-performing older adults”)—individuals aged 80 years or older who retain exceptional cognitive and memory performance equal to or greater than that of individuals aged in their 50s or 60s [424]—had a higher level of education [425], a significantly thicker brain cortex [426], and a greater anterior cortex volume [427] compared with their age-matched peers with average-for-age memory and cognition (*this is particularly important since the anterior cortex is linked with the phenomenal first-person perspective and the sense of agency or being a self [315]*). Furthermore, long-term smoking has been associated with brain aging and degeneration [205,428,429]. The findings on alcohol consumption are rather mixed: while it was shown that heavy drinking is associated with a greater loss of grey and white matter in the brain [430] and with brain aging [431], moderate alcohol consumption (in particular wine) may be beneficial for the cardiovascular system, which is related to brain health and is associated with a reduced risk of dementia and better cognitive function [432]. Such positive effects might be mediated by polyphenols, flavonoids, and organic acids present in wine, which have antioxidant, anti-inflammatory, and neuroprotective mechanisms [433]. Interestingly, it has been documented recently that wine consumption is associated with a decelerated epigenetic aging [276]. The larger proportion of left-handed individuals in the BBA < CA subgroup (see Table 2) is peculiar and requires further study; however, some clues in the literature may already be established. Left-handed individuals usually experience a very quick reversal of pathological states, including brain functions after trauma or disorders [434]; left-handedness may be associated with a longer lifespan, especially if one reaches middle age [435,436], and there are disproportionately fewer left-handed patients with Alzheimer’s disease [437,438]. All this may point to some potential neuroprotective mechanisms present in left-handed individuals.

Pre-intervention BBA score could be a covariate that may contribute differently to the overall results of the present study and, hence, we examined the effects of both interventions on the BBA separately after splitting the whole sample based on pre-intervention BBA scores (Figure 5). We found that in the experimental/nutraceuticals group, for the participants whose pre-intervention BBA was older than their CA, the BBA scored, on average, 6.77 years younger at the endpoint of the intervention compared to the beginning. For the participants whose pre-intervention BBA was younger than their CA, the BBA scored, on average, only 0.64 years younger at the endpoint of the intervention compared to the beginning (Figure 5). These results indicate that the BBA reversal after nutraceuticals supplementation was stronger for participants whose pre-intervention BBA was older than their CA (*a result that is consistent with a recent finding that supplementation with alpha-ketoglutarate and vitamins resulted in a stronger decrease in systemic biological age in biologically older individuals [416]*). A straightforward explanation could be that individuals with initially younger brains (and thus high brain and cognitive reserves) are already functioning at an optimal level (see also [260]). Consequently, additional interventions do not further optimize the functional brain patterns (contributing to the BBA) because of a ceiling effect: both capacity and efficiency in their brains have already reached the limit and “topped out”. As for the control/lifestyle group, the BBA scored, on average, 0.25 years older (for those whose pre-intervention BBA was older than their CA) and 0.13 years younger (for those whose pre-intervention BBA was younger than their CA) at the end of the intervention when compared with the baseline (Figure 5). Both changes were small and nonsignificant; therefore, one may conclude that the lifestyle intervention was not effective for reversing BBA but rather stabilized it despite the pressure of CA, thus achieving age deceleration.

It is known that there are sex-related differences in brain structure (thickness of the cortex and proportion of grey matter) [439], morphology [440], functional organization [441], and aging trajectories [207] in humans. Therefore, we considered sex as a covariate that may affect the overall results and, hence, examined sex-specific differences in BBA for both interventions. For both females and males, the BBA scored younger at the end of the nutraceutical supplementation when compared with the baseline; however, the decrease in the BBA scores (i.e., age reversal) was stronger for females (Figure 4). This discrepancy between sexes may relate to the persistent observation that age-related brain atrophy (or metabolic brain age) is more extensive in males than in females [442,443] and, thus, initially, the BBA could be older in males when compared with females. Indeed, the BBA in males at the beginning of the study was, on average, 52.58 years old, while in females it was 44.31 years old, thus signifying an older brain BA in males at the pre-intervention time-point when compared with females. By comparison, for the lifestyle intervention, the BBA decreased slightly only in males, while it increased insignificantly in females at the end of the intervention (Figure 4). Both changes were very small, and knowing that lifestyle changes mostly stabilize the BBA (i.e., deceleration of aging; see above), one may conclude that the lifestyle intervention did not cause any significant changes in the BBA of both sexes, it only kept the BBA rate slowed down despite the pressure of CA. Additionally, other influences, such as genetic variations in females and males, may have had a further impact on the BBA [444,445].

## 5. Conclusions, Significance, Limitations, and Future Research

The present study demonstrated that brain BA deceleration, and even reversal, with accompanying improvements in mental–physical health comorbidities is possible in humans using accessible interventions, such as lifestyle changes or nutraceutical supplementation, within a practical time frame (~13 months). Although much more remains to be investigated, from a translational perspective, these findings are noteworthy given that lifestyle changes such as calorie restriction and intermittent fasting, physical exercise and proper sleep, and vitamins and nutrients are the most commonly used practices worldwide. While currently there is an unprecedented explosion of breakthroughs in many areas of basic science and even translational medicine related to aging [96,308], the new putative interventions are unlikely to be available to the average person any time soon. Therefore, such commonly available and relatively affordable interventions as a healthy lifestyle and nutraceutical supplementation are extremely important. Indeed, if these interventions can be made practical and scalable, we may find ourselves in a future in which we have “no time to age” [74].

**Figure 7:**
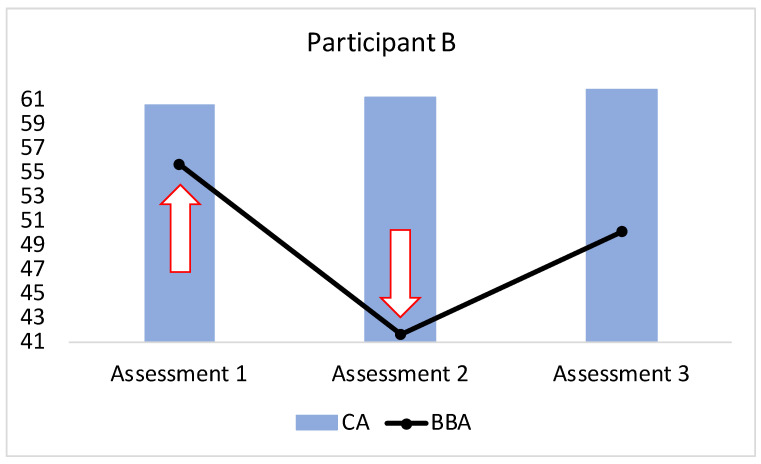
Intervention-induced changes in BBA as a function of nutraceutical supplementation across three assessments. The *y*-axis represents years for the BBA and CA. CA: chronological age; BBA: biological brain age. The arrow pointing up indicates the start of the intervention, and the arrow pointing down indicates the end of the intervention.

Despite the remarkably promising results, the present study has several limitations. While this study was not small, larger prospective trials are warranted to confirm the initial observations of the present retrospective study. Furthermore, both interventions (experimental and active control) contained several components individually adjusted for every participant, and this was considered an advantage. At the same time, this makes it impossible to attribute the overall beneficial result of the intervention to any individual element of the intervention. As for nutraceutical compounds, currently there are new substances emerging that have a putatively strong anti-aging and geroprotective potential by targeting multiple hallmarks of aging [9] (see also [49,416]). Such new substances may be considered in future studies of BBA. Another limitation of the present study relates to the absence of an evaluation of the sustainability of BBA changes following some temporal interval after the discontinuation of the interventions to see if the decrease in BBA remains stable or rebounds back towards the pre-intervention levels. However, hints are already possible to derive; for example, we had one participant who had three assessments instead of two, roughly 6 months apart. At the first assessment, the person received the nutraceutical supplementation program, which she followed for 6 months until the second assessment, when the program was discontinued; the third assessment was conducted after another 6 months, without any intervention (Figure 7). For this person, although their pre-intervention BBA was lower than their CA (assessment 1), the BBA noticeably decreased postintervention after 6 months (assessment 2) and then had a tendency to rebound back towards the pre-intervention level 6 months after discontinuing the intervention (assessment 3). At the same time, the BBA rebound was not complete, still having an improvement of 11.6 years in comparison with their CA at the third assessment. If future durability studies do establish a gradual loss of BBA reversal compared to baseline, it will be interesting to determine whether periodic repetition of the intervention might restore the initially achieved level of BBA reversal. It also remains to be seen whether further adjustments of the combination of nutraceutical compounds and/or their dosages will further augment BBA reversal. Additionally, the combination of nutraceutical supplementation and lifestyle changes within the same intervention program may be even more impactful, and this remains to be studied in future trials (for example, see [446]). Furthermore, since this was the first study to show that nutraceutical supplementation and lifestyle could affect brain aging, only standard statistical analyses were performed, resulting in a large spectrum of results. However, secondary analysis of these data will be needed to dissect the causal relationships between the BBA rates and nutraceuticals/lifestyle factors and to estimate the potential correlations between the covariates. Additionally, we applied a previously developed method to quantify the qEEG-derived BBA. At the same time, there may be other methods, and they may produce different results. Yet another limitation relates to the potential role of hormones which may contribute to changes in BBA, as the levels of hormones are naturally different in males and females, as well as in young and older persons. The hormonal status was not controlled in the present study and, therefore, future research should consider it. CA, itself, could also be a confounding factor, and future larger prospective studies should specifically address this issue, though it has been documented previously that lifespan extension is comparable if the anti-aging intervention is initiated at a young age, middle age, or in late life [354]. Finally, the participants were self-selected with respect to the intervention type (nutraceutical compounds vs. lifestyle), and the blinding of interventions was not possible due to the different nature of the selected interventions. At the same time, the participants were blinded to the interventions’ *primary output related to the qEEG-derived BBA* (participants thought that the interventions aimed to improve their general well-being); therefore, a potential placebo effect related to BBA in both interventions could be excluded. In this respect, this retrospective study can be considered single-blind.

In spite of these limitations, some of the strengths of our study include a relatively large sample size (42 participants in the experimental/nutraceuticals group and 47 in the control/lifestyle group), a wide range of CAs in the sample spanning from 25 to 77 years old, and the use of the qEEG-derived BBA as a simple and reliable biomarker of brain aging. Furthermore, the present study had a longitudinal design, which allowed for conclusions regarding the directionality of the anti-aging intervention effects.

## Figures and Tables

**Table 2:** Demographic and clinical data for the subgroups with the pre-intervention BBA older or younger than the CA.

Characteristics	BBA > CA	BBA < CA	*p*-Value	Test Type
Sample size (N)	28	61	Not applicable	Not applicable
Sex (% of females)	57.1	65.6	Not significant	Chi-square
Chronological age—CA (mean/st.d)	42.2 (8.9)	52.7 (10.7)	0.00001	Mann–Whitney U test
Brain biological age—BBA (mean/st.d)	51.4 (7.3)	37.3 (9.9)	0.00001	Mann–Whitney U test
Brain resources—BR (mean %/st.d)	−10.5 (9.2)	+18.6 (11.0)	0.00001	Mann–Whitney U test
Healthy lifestyle habits (% of those who have)	14.3	14.8	Not significant	Chi-square
Current health symptoms (% of those who have)	42.9	60.7	0.010846	Chi-square
Past health problems (% of those who had)	53.6	62.3	Not significant	Chi-square
Relatives with mind/brain disorders (% of those who have)	17.9	23.1	Not significant	Chi-square
Anxiety–Beck (mean/st.d)	7.0 (6.5)	8.5 (6.8)	Not significant	Mann–Whitney U test
Anxiety–Ham (mean/st.d)	7.3 (5.8)	9.2 (6.0)	Not significant	Mann–Whitney U test
Depression–Beck (mean/st.d)	5.6 (5.3)	6.6 (5.9)	Not significant	Mann–Whitney U test
Big-5—neuroticism (mean/st.d)	2.7 (0.8)	2.9 (0.7)	Not significant	Mann–Whitney U test
Handedness (% of right-handed)	92.8	83.6	0.046061	Chi-square
Marital status (% of married)	66	82	0.0099	Chi-square
Marital status (% of divorced)	19.7	11.5	Not significant	Chi-square
Marital status (% of single)	14.6	6.5	0.037897	Chi-square
Education (% of those who have a PhD)	3.7	16.4	0.004678	Chi-square
Education (% of those who graduated from university or institute)	67.8	75.4	Not significant	Chi-square
Education (% of those who completed high school (≥11–12 years))	28.5	8.2	0.000131	Chi-square
Job (% of directors or CEOs)	28.6	21.3	Not significant	Chi-square
Job (% of senior managers)	17.9	23.1	Not significant	Chi-square
Job (% of junior managers)	46.4	54	Not significant	Chi-square
Job (% of students or trainees)	7.1	1.6	Not significant	Chi-square
Number of interests or hobbies (mean/st.d)	3.0 (1.3)	4.5 (1.6)	0.00026	Mann–Whitney U test
Smoking (% of those who smoke)	7.1	1.2	0.030383	Chi-square
Alcohol consumption (1–2 drinks per week; %)	46.4	47.5	Not significant	Chi-square
Alcohol consumption (3–4 drinks per week; %)	35.7	34.4	Not significant	Chi-square
Alcohol consumption (5–7 drinks per week; %)	14.3	6.6	Not significant	Chi-square
Alcohol consumption (8–10 drinks per week; %)	3.6	11.5	0.037056	Chi-square

The notes are the same as in Table 1.

**Table 3:** Current health symptoms (% of participants who have).

Groups	Pre-Intervention	Postintervention
Experimental/nutraceuticals	33.3	4.7
Control/lifestyle	40.2	32

## Data Availability

Not applicable.
